# WHO definitions for reserve antibiotics

**DOI:** 10.2471/BLT.25.293893

**Published:** 2025-11-12

**Authors:** Veronica Zanichelli, Jessica Bartoszko, Benedikt Huttner, Mark Loeb, Nicola Magrini, Marc Mendelson, Mike Sharland, Deborah Tong, Loice Achieng Ombajo, Lorenzo Moja

**Affiliations:** a Medicines and Health Products Policy and Standards, World Health Organization, Avenue Appia 20, 1211 Geneva, Switzerland.; b Department of Health Research Methods, Evidence and Impact, McMaster University, Hamilton, Canada.; c Department of Antimicrobial Resistance, World Health Organization, Geneva, Switzerland.; d WHO Collaborating Centre for Evidence-Based Research Synthesis and Guideline Development, Bologna, Italy.; e Division of Infectious Diseases and HIV Medicine, Department of Medicine, University of Cape Town, Cape Town, South Africa.; f Centre for Neonatal and Paediatric Infections, St. George’s, University of London, England.; g Department of Clinical Medicine and Therapeutics, University of Nairobi, Nairobi, Kenya.

The World Health Organization (WHO) Access, Watch, Reserve (AWaRe) classification of antibiotics is closely linked to the essential medicines concept and the *WHO Model lists of essential medicines*, which inform national essential medicines lists.^1^^,^^2^ These national lists are a fundamental strategy in health-care systems to ensure effective use of constrained resources. Furthermore, by focusing on essential antibiotics, health-care systems can better meet the health needs of their populations and mitigate the risk of antibiotic resistance.

The AWaRe classification categorizes antibiotics into three groups: Access, Watch or Reserve. This classification, developed in 2017 by the Expert committee on the selection and use of essential medicines, has a twofold objective: prioritizing the most important antibiotics for human health; and supporting stewardship and surveillance activities.^1^ As of 2025, WHO has assigned 268 antibiotics used in humans to one of the AWaRe groups.

Since 2019, the expert committee defines reserve antibiotics as last-resort antibiotics that are only to be used in patients with confirmed or suspected life-threatening infection due to multidrug-resistant (MDR) bacteria.^3^ The AWaRe classification also states that reserve antibiotics should only be used when other therapeutic alternatives have failed or are unsuitable.^3^ Finally, only reserve antibiotics with proven activity against critical or high priority pathogens identified by the *WHO priority pathogens list* can qualify for inclusion on the WHO essential medicines list (Box 1).^3^ The 2025 WHO essential medicines list includes 50 antibiotics, 10 of which are in the reserve group. A further 20 antibiotics are also currently classified as reserve for surveillance and stewardship activities, although they are not on the WHO essential medicines list.

Box 1 **Essential reserve antibiotics and their current definition**

Last-resort antibiotics with proven activity against critical or high-priority pathogens identified by the *WHO bacterial priority pathogens list* to be used only for highly selected patients with confirmed or suspected life-threatening infections due to MDR bacteria. They should be only used when other therapeutic alternatives have failed or are unsuitable. To ensure their continued effectiveness, they should be closely monitored and prioritized as targets of stewardship programmes.

The *WHO Model list of essential medicines 2025* includes 10 reserve antibiotics: cefiderocol, ceftazidime + avibactam, ceftolozane + tazobactam, parenteral colistin and polymyxin B, fosfomycin, meropenem + vaborbactam, plazomicin, linezolid and tedizolid.

AWaRe is now the globally accepted method for classifying antibiotics. In 2024, all United Nations (UN) Member States committed to the target that, by 2030, 70% of all human antibiotic use consists of access antibiotics.^4^ The AWaRe classification is also gaining momentum outside the UN, as evidenced by its use to interpret antibiotic use in research studies,^5^ surveillance reports by public health agencies^6^ and the *Global antimicrobial resistance and use surveillance system* (known as GLASS).^7^

AWaRe was designed as a dynamic tool, intended to evolve with new evidence and real-world user experience. While its uptake across health systems is encouraging, the current definition and selection criteria for reserve antibiotics in the WHO essential medicines list would benefit from targeted revisions to address existing limitations (Box 2). The proposed revisions are based on deliberations within the WHO Essential Medicines List Secretariat, the WHO Technical Advisory Group on AWaRe and consultations with other subject-matter experts.

Box 2 **Elements of the 2025 essential reserve antibiotic definition where changes are possible (order not indicative of priority)**

- Level of evidence needed to demonstrate proven activity against critical or high priority pathogens identified by the *WHO bacterial priority pathogens list* is not always clear;^8^
- The relationship between the current definition and the *WHO bacterial priority pathogens list* (both the 2017 and 2024 editions) is not univocal;^8^ for example, some antibiotics with consistent in vitro activity against priority pathogens are not classified as reserve;
- Evidence to develop guidance on the appropriate empiric use of reserve antibiotics to treat life-threatening infections due to suspected MDR bacteria is limited;^a^
- The definition for MDR bacteria has long been debated but a single, universally accepted definition, is still lacking;^a^
- Use of the same principles for selecting all antibiotics on the WHO essential medicines list regardless of AWaRe classification; for example, reserve antibiotics might deserve different criteria (such as parsimony criterion valid for access and watch antibiotics could not apply to reserve antibiotics);
- WHO innovation criteria for novel antibiotics, and research and development antibacterial pipeline are not explicitly considered;^a,^^9^ and
- Access issues and strategies to increase access are not considered.

AWaRe: Access, Watch, Reserve; MDR: multidrug resistant; WHO: World Health Organization.^a^ Elements open to reflection that also apply to reserve antibiotics in general (that is, also to reserve antibiotics not included in the WHO essential medicines list).

For essential reserve antibiotics, the statement "proven activity against critical or high priority pathogens identified by the *WHO priority pathogens list*"^3^ poses problems. First, the level of evidence demonstrating activity against priority pathogens, whether in vitro or in humans, required to include a reserve antibiotic on the WHO essential medicines list is unclear. Furthermore, not all infections caused by these priority pathogens require a reserve antibiotic to be effectively treated. Watch antibiotics such as meropenem and vancomycin may be appropriate to treat infections caused by third-generation cephalosporin-resistant *Enterobacterales* (critical priority) and methicillin-resistant *Staphylococcus aureus* (high priority), respectively. In other words, the statement "proven activity against critical or high priority pathogens"^3^ is not exclusive to reserve antibiotics. The *WHO bacterial priority pathogens list* was originally developed to guide research and development of new antibiotics, not WHO essential medicines list decisions or antimicrobial stewardship. Therefore, this statement may need to be reviewed.^8^

High-quality clinical evidence for empiric use is lacking for most reserve antibiotics. In its absence, inappropriate use drives resistance; for example, the prevalence of colistin resistance in *Klebsiella pneumoniae* has been steadily increasing in China as in European countries,^10^ and increasing resistance could also compromise the efficacy of newer reserve antibiotics.^11^ Evidence for empiric use is challenging to develop, but is critical when considering treatment for patients with life-threatening but unconfirmed MDR bacterial infections and to reduce the incidence of resistance to these last-resort antibiotics. Given the lack of high-quality clinical evidence and differences in local epidemiology, the Infectious diseases society of America provides pragmatic guidance on how to treat antimicrobial-resistant gram-negative infections, based on comprehensive reviews of the literature, clinical experience and expert opinion.^12^ Although primarily focused on the targeted treatment of confirmed MDR infections, this guidance also includes a brief section on empiric treatment, providing practical recommendations. Guidance is also provided in the AWaRe antibiotic book for essential reserve antibiotics^13^ and could be expanded, based on a similar pragmatic approach. Finally, in the statement "confirmed or suspected life-threatening infections due to MDR bacteria",^3^ it is unclear that the term confirmed or suspected refers to the presence of MDR bacteria, rather than life-threatening infection.

In outlining reserve antibiotics, MDR is not clearly defined, and consensus on a standardized, internationally recognized definition has yet to be reached. Definitions for MDR, extremely (or extensively) drug-resistant, pandrug-resistant and difficult-to-treat resistance have been proposed for select bacteria;^14^ however, their use is not widespread in practice. This long-standing debate might not have direct implications for the essential reserve antibiotic definition since the principle of multiple resistance is inherently more significant for their interpretation and subsequent actions. Nevertheless, efforts to refine the MDR definition, particularly for gram-negative bacteria, may be justified to standardize surveillance, guide clinical management, inform public health policies and help prioritize the development of new antibiotics.

Decisions to list reserve antibiotics on the WHO essential medicines list are made by the expert committee based on agreed principles that apply to all AWaRe group antibiotics such as benefits, harms and parsimony, that is, not listing too many antibiotics with a similar efficacy and safety profile.^1^ Despite these guiding principles, standardizing applications for new reserve antibiotics would be beneficial, with evidence sources extending beyond pivotal randomized trials. Real-world data can provide further information on benefits (such as increased adherence due to ease of administration, superior effectiveness compared to older agents) and harms (for example increased incidence of resistance, side-effects).

The AWaRe classification and decisions to list reserve antibiotics on the WHO essential medicines list can have implications for the research and development of novel antibiotics, and vice versa. Every two years, the WHO research and development antibacterial pipeline is updated to reflect the landscape of antibiotics in clinical development.^9^ Furthermore, WHO has indicated four innovation criteria for novel antibiotics: (i) no cross resistance to other antibiotic classes; (ii) new chemical class; (iii) new target; and (iv) new mode of action.^9^ Assessing which, and whether, innovation criteria are addressed by reserve antibiotics, including those on the WHO essential medicines list, may contribute to identifying unmet health needs and gaps in innovation that could be considered essential to address. For example, vaborbactam, approved in combination with meropenem, represents a new chemical class, is classified as reserve, and is the only agent since 2017 that met at least one of the innovation criteria and was subsequently included on the list. A future revision of the reserve definition may consider whether these innovation criteria have a critical role in the AWaRe classification and WHO essential medicines list selection principles.

The definition mentions last resort, emphasizing the life-saving role of reserve antibiotics. In low-resource settings, carbapenems or vancomycin (Watch antibiotics) could be the only available last-resort options. Against this scenario, the reserve definition is primarily aligned with high-income countries. Access to reserve antibiotics is not the same across jurisdictions and is especially limited in low-resource settings.^7^ A future iteration of the definition may place value on access as this dimension is likely to have important implications for low- and middle-income countries. To ensure patients globally benefit from essential reserve antibiotics, comprehensive implementation strategies, that direct the path to increased equitable access and to appropriate use with diagnostic support, need to follow. One such example is SECURE, a collaboration between the Global Antibiotic Research and Development Partnership and WHO, that seeks to expand access to essential antibiotics and ensure their appropriate use.^15^

Highlighting current areas where the definition of essential eserve antibiotics may need revision could help inform corresponding changes to the selection of reserve antibiotics for inclusion in the WHO essential medicines list. In 2025, the expert committee considered a revision of the current AWaRe definitions and criteria for inclusion of AWaRe antibiotics on the list. However, given the complexity of the issue and its implications, the expert committee decided to postpone a decision and to first conduct a structured review of AWaRe categories. This structured review will involve broad stakeholder consultation including the technical advisory group, and a comprehensive assessment of the available evidence, with the intention of submitting an application to revise the reserve antibiotic definition, and listed reserve antibiotics, for consideration at the next essential medicines list expert committee meeting in 2027. Therefore, the expert committee did not propose any immediate changes to the reserve definition and to criteria for their inclusion in the WHO essential medicines list. Any future proposed changes in the reserve definition will be open to public consultation to capture expectations on standardization and evolutions of AWaRe.
